# The Mediating Role of WBC in the Relationship Between Triglyceride–Glucose Index and Chronic Pain: Evidence From NHANES 2001–2004 Data

**DOI:** 10.1155/prm/3793191

**Published:** 2026-04-21

**Authors:** Di Zhou, Fan Jin, Ling Zhang, Jin Chen, Lv Tian, Jie Li

**Affiliations:** ^1^ Department of Hepatobiliary and Pancreatic Surgery, Zhuji People’s Hospital, Zhuji, Zhejiang, China; ^2^ Department of Anesthesiology, Zhuji People’s Hospital, Zhuji, Zhejiang, China; ^3^ Department of Cardiothoracic Surgery, Zhuji People’s Hospital, Zhuji, Zhejiang, China; ^4^ Department of Neurology, Zhuji Affiliated Hospital of Wenzhou Medical University, Zhuji, Zhejiang, China; ^5^ Department of Neurology, The First Affiliated Hospital of Lishui University & Lishui People’s Hospital, Lishui, Zhejiang, China

**Keywords:** chronic pain, mediator analysis, NHANES, TyG index, white blood cell

## Abstract

**Background:**

The relationship between triglyceride–glucose (TyG) index and chronic pain remains poorly understood. This study sought to evaluate how the TyG index relates to both the occurrence of chronic pain and all‐cause mortality in affected individuals, while also assessing whether white blood cell (WBC) count played an intermediary role in these associations.

**Methods:**

We analyzed data from 10,452 adults (aged ≥ 20 years) in the National Health and Nutrition Examination Survey (NHANES) from 2001 to 2004. Weighted multivariable logistic regression assessed the TyG index–chronic pain association, with mediation analysis evaluating the role of WBC count. Cox regression analyzed the predictive role of the TyG index for survival in chronic pain patients, and potential mediation analysis examined the influence of WBC.

**Results:**

Among 3764 analyzed participants, the TyG index demonstrated significant associations with chronic pain in both continuous (OR = 1.25, 95% CI = 1.04–1.49, *p* = 0.020) and categorical models (Q5 vs. Q1: OR = 1.58, 95% CI = 1.09–2.31, *p* = 0.020) after full adjustment. WBC potentially mediated 9.0% (95% CI = 2.6%–12.4%) of this association. In the mortality analysis, the elevated TyG index predicted increased all‐cause mortality risk (T3 vs. T1: HR = 2.05, 95% CI: 1.46–2.89, *p* < 0.001) in chronic pain patients, and WBC potentially mediated 12.0% (95% CI: 1.0%–23.1%) of this association.

**Conclusions:**

The TyG index was independently associated with both chronic pain and increased mortality in chronic pain patients. Systemic inflammation partially mediated these links, suggesting that inflammation was a potential pathway connecting insulin resistance, chronic pain, and survival outcomes.

## 1. Introduction

Chronic pain has emerged as a major global health concern, characterized by its widespread prevalence, substantial contribution to the disability burden, and a profound impact on both physical and psychological health [1]. Chronic pain was characterized by prolonged or intermittent pain extending beyond 3 months [2]. Research indicated a significant global prevalence, with approximately 20% of adults experiencing chronic pain annually, and roughly 10% receiving a formal diagnosis [3]. Research conducted over 4 years in the United Kingdom found that chronic pain developed annually in 8.3% of individuals, while the likelihood of complete resolution was 5.4% [4]. Chronic pain has significant implications for both physical and mental health, resulting in comorbidities such as depression, anxiety, and sleep disturbances [5, 6]. These detrimental effects on quality of life underscored the critical importance of identifying and understanding the risk factors associated with chronic pain development.

The scientific literature continues to uncover important relationships between insulin resistance (IR) and pain disorders. Clinical investigations have revealed significant connections, with one cross‐sectional study identifying a relationship between IR and central pain mechanisms in fibromyalgia patients [7], and that the triglyceride–glucose (TyG) index serves as a reliable biomarker for predicting chest pain episodes [8]. What’s more, in Zucker diabetic fatty rat models, researchers demonstrated a bidirectional relationship between IR and chronic pain [9]. However, the extent of this association in humans remains underinvestigated, highlighting the need for comprehensive studies to clarify this relationship and guide potential therapeutic interventions.

The TyG index, derived from fasting triglyceride and glucose measurements, serves as a readily available, inexpensive, and dependable biomarker for evaluating IR. Its simplicity and reliance on routine laboratory values contributed to its widespread application in clinical and research settings [10].

Building upon these scientific foundations, the current study employed data from the National Health and Nutrition Examination Survey (NHANES) 2001–2004 to investigate two primary aspects: (1) examining whether the TyG index elevation correlates with both chronic pain occurrence and mortality risk in affected individuals and (2) evaluating white blood cell (WBC) count as a potential mediator in the TyG–pain and TyG–mortality relationships. This dual‐focused methodology offers novel perspectives on metabolic‐pain pathophysiology.

## 2. Materials and Methods

### 2.1. Participates

The NHANES implemented a sophisticated, biennial stratified sampling methodology to ensure nationally representative estimates of the US population’s health and nutritional status. The study protocol received National Center for Health Statistics Ethics Review Board approval (No. Protocol#98‐12), and all participants provided written informed consent, ensuring adherence to ethical research standards. NHANES collected comprehensive data through multiple modalities, including detailed demographic information, standardized survey responses, physical examinations, and laboratory analyses.

For the current analysis, we initially identified 10,452 eligible participants aged 20 years or older from the NHANES 2001–2004 dataset who had completed the Miscellaneous Pain Questionnaire. Participants were excluded in a stepwise manner according to the following criteria:

1. Missing or denied pain data (*n* = 10).
2. Missing TyG index data (*n* = 5979).
3. Missing data on other key covariates (*n* = 699), including diabetes mellitus (DM) (*n* = 242), waist circumference (*n* = 162), serum iron (*n* = 8), smoking status (*n* = 4), body mass index (BMI) (*n* = 49), WBC (*n* = 2), lymphocyte (LYM) (*n* = 13), alcohol status (*n* = 211), education (*n* = 3), and cancer (*n* = 5).

**FIGURE 1:**
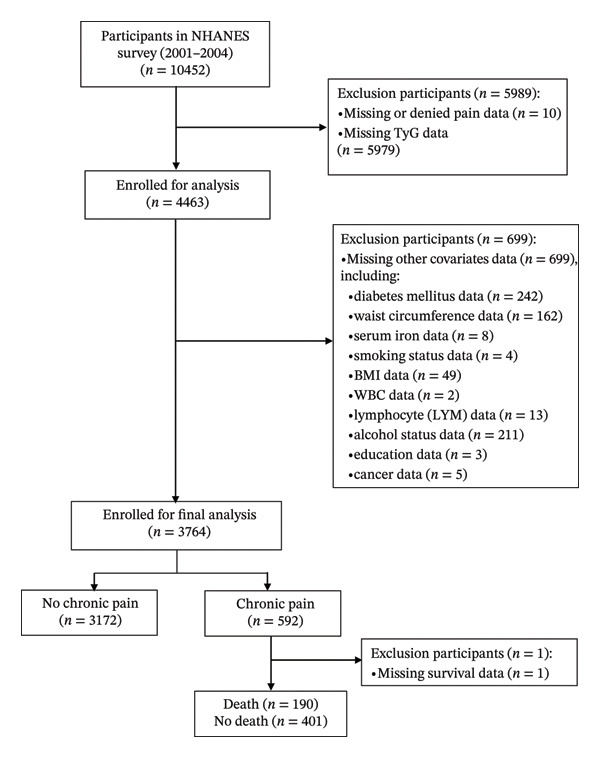
Flowchart of the study population.

The above exclusion steps resulted in a final analytical cohort of 3764 participants for the cross‐sectional analysis of chronic pain (Figure 1).

Additionally, in the subsequent cohort analysis of survival outcomes among participants with chronic pain, one individual was further excluded due to missing survival data (*n* = 1) (Figure 1).

To assess whether missing TyG‐related data introduced systematic bias, we compared baseline demographic and clinical characteristics between participants with complete TyG data and those with missing TyG values. As shown in Supporting Table S2, most baseline features did not differ significantly between the two groups, suggesting that the missing data mechanism was consistent with missing completely at random (MCAR). This implies that the missingness was not substantially associated with measured or unmeasured covariates in this study [11].

### 2.2. Primary Exposure and Outcome Measures

The primary exposure variable in this investigation was the TyG index, which was derived through the following formula: TyG index = Ln [fasting triglycerides (mg/dL) × fasting glucose (mg/dL)]/2 [12].

The primary outcome, chronic pain status, was determined using the standardized McGill Pain Questionnaire (MPQ), specifically Items MPQ100 and MPQ110. The presence of pain lasting more than 24 h in the past month was determined using the MPQ100 item. The duration of this pain was subsequently evaluated through the MPQ110 question: “How long have you experienced this pain?” Participants reporting pain (MPQ100 = 1) with a duration of at least three months (MPQ110 = 3 or 4) were categorized as having chronic pain. Those who either denied experiencing pain in the past month (MPQ100 = 2) or reported pain lasting less than three months (MPQ100 = 1 with MPQ110 = 1 or 2) were classified as not having chronic pain. Participants who did not provide a response or answered “I do not know” to either question were excluded from the analysis due to missing data [3, 13].

### 2.3. Definition of Mortality

Survival analyses were performed to evaluate TyG index–associated mortality risk among chronic pain patients. Participant outcomes were determined through linkage with the National Death Index records (through December 31, 2019), utilizing the “MORTSTAT” variable for mortality status and “PERMTH_EXM” for follow‐up period calculation (https://www.cdc.gov/nchs/data-linkage/mortality.htm).

### 2.4. Covariates

Covariates were divided into three groups: demographic data, basic diseases, and laboratory examinations. Demographic data included age, sex, BMI, smoking status [13] (categorized as: never smoker [< 100 cigarettes lifetime], former smoker [> 100 cigarettes lifetime but currently abstinent], or current smoker [> 100 cigarettes lifetime and currently smoking]), alcohol consumption [15] (classified as: never [< 12 drinks lifetime], mild [≤ 1 drink/day for women, ≤ 2 drinks/day for men], moderate [2 drinks/day for women, 3 drinks/day for men or binge drinking 2–4 days/month], heavy [≥ 3 drinks/day for women, ≥ 4 drinks/day for men or binge drinking ≥ 5 days/month], or former [≥ 12 drinks/year but abstinent in the previous year]), and education level (stratified as: under high school, high school or equivalent, or above high school).

Basic diseases included chronic kidney disease (CKD) (Yes/No), cardiovascular disease (CVD) (Yes/No), hyperlipidemia (Yes/No), DM [16] (categorized as DM [DM: HbA1c > 6.5% or fasting glucose > 126 mg/dL or antidiabetic medication use], impaired fasting glucose [IFG: HbA1c 5.7%–6.4% or fasting glucose 100–125 mg/dL], or normal [HbA1c < 5.7% and fasting glucose < 100 mg/dL]), cancer (Yes/No), and anemia (Yes/No).

Laboratory examinations included neutrophil–lymphocyte ratio (NLR), systemic immune inflammation index (SII), WBC, LYM, monocytes (MON), neutrophils (NEU), fasting glucose, fasting triglycerides, red cell distribution width (RDW), albumin, alanine aminotransferase (ALT), serum iron, and hemoglobin (HB).

### 2.5. Statistical Analysis

We incorporated the NHANES fasting subsample weight (“WTSAF2YR”) to account for the complex survey design and ensure nationally representative estimates. Detailed information regarding the survey methodology and weight calculation procedures is available through the official NHANES analytic guidelines (https://wwwn.cdc.gov/nchs/nhanes/analyticguidelines.aspx).

Participants were stratified by chronic pain status for baseline comparisons. Normally distributed continuous measures were reported as mean ± SE and assessed via *t*‐tests, while nonparametric variables underwent Wilcoxon rank‐sum testing. Categorical data, expressed as weighted frequencies (percentages), were evaluated using *χ* ^2^ tests.

Weighted logistic regression with sequential covariate adjustment was conducted to assess the association between the TyG index and chronic pain prevalence. To examine the detailed, nonlinear dose–response relationship between TyG and chronic pain, TyG was modeled both as quintiles and as a continuous variable, allowing for the detection of potential threshold effects. OR estimates (95% CIs) were derived from the following: Model 1 (unadjusted), Model 2 (adjusted for age, sex, and education), Model 3 (adjusted for age, sex, education, CVD, hyperlipidemia, albumin levels, NLR, serum iron, HB, smoking status, alcohol status, cancer, anemia, CKD, BMI, and DM), and Model 4 (adjusted for age, education, sex, NLR, smoking status, cancer, CKD, BMI, and DM, according to correlation analysis [VIF < 10, and age], Table S3A).

Subgroup analyses were performed to assess effect modification across key demographic and clinical variables: age groups (< 60 vs ≥ 60 years), sex, BMI categories (< 30 vs. ≥ 30 kg/m^2^), educational attainment (below high school, high school/equivalent, or above high school), diabetes status (nondiabetic, IFG, or DM), and hyperlipidemia (yes or no). Stratified logistic regression models, adjusted according to Model 3, were utilized. Likelihood ratio tests were subsequently performed to assess significant differences and interactions across the defined subgroups. Bonferroni correction was performed to adjust the *p* value.

Mediator analysis, adjusted for covariates as in Model 4, was performed to evaluate WBC count or NLR count as a potential mediator in the TyG index–chronic pain relationship. We also evaluated WBC count as a potential mediator in the TyG index–mortality relationship in chronic pain patients. This involved estimating the overall effect (α), the indirect effect (β1), and the indirect effect (β2).

In the Cox regression, to ensure a sufficient number of mortality events within each category and to obtain more stable hazard ratio (HR) estimates with narrower confidence intervals (CIs), the TyG index was modeled both as tertiles and as a continuous variable, allowing for the detection of potential threshold effects. The mortality risk associated with the TyG index was assessed using Cox proportional hazards regression (expressed as adjusted HRs with 95% CIs), supplemented by Kaplan–Meier survival curves stratified by TyG tertiles. HR estimates (95% CIs) were derived from Model 1 (unadjusted), Model 2 (adjusted for age, sex, and education), Model 3 (adjusted for age, sex, education, CVD, hyperlipidemia, albumin levels, NLR, serum iron, HB, smoking status, alcohol status, cancer, CKD, BMI, and DM), and Model 4 (adjusted for age, education, sex, CVD, albumin, HB, NLR, alcohol status, CKD, BMI, and DM, according to correlation analysis [VIF < 10, and age], Table S3B). The proportional hazards assumption for Model 4 was tested using Schoenfeld residuals.

To assess the robustness of our findings to missing data, we performed a sensitivity analysis using multiple imputation by chained equations (MICE) for covariates with missing values. The imputation model included all covariates used in the primary analyses. Each imputed dataset was analyzed using the same multivariable models as in the primary analysis, with the TyG index, chronic pain status, and survival outcomes remaining unimputed.

Potential curvilinear associations were investigated via restricted cubic spline (RCS) analysis.

The analytical significance threshold was established at *p* < 0.05 (two‐tailed), implemented using R programming environment (Version 4.3.1).

## 3. Results

### 3.1. Cross‐Sectional Analysis

#### 3.1.1. Basic Characteristics

The analytical cohort comprised 3764 participants (51.7% male, *n* = 1945), with 15.7% (*n* = 592) meeting the criteria for chronic pain, which was consistent with recent estimates from other NHANES‐based studies [17, 18] and general population estimates [19]. Comparative analysis revealed significant demographic and clinical differences between groups. Chronic pain patients were typically older, less educated, and had higher BMI, smoking rates, and alcohol use. They also exhibited elevated metabolic markers (WBC, triglycerides, waist circumference, and TyG index) and higher rates of diabetes and CVD (Table 1).

**TABLE 1:** Baseline characteristics of participants with or without chronic pain.

Variables	Overall (*n* = 3764)	No pain (*n* = 3172)	Chronic pain (*n* = 592)	*p* value
Age, years	46.227 (0.550)	45.747 (0.630)	48.525 (0.745)	0.006
Sex, *n* (%)				0.010
Female	1819 (49.626)	1485 (48.263)	334 (56.157)	
Male	1945 (50.374)	1687 (51.737)	258 (43.843)	
BMI, kg/m^2^	28.151 (0.133)	27.927 (0.146)	29.223 (0.311)	< 0.001
CVD, *n* (%)	430 (8.035)	311 (6.330)	119 (16.205)	< 0.001
CKD, *n* (%)	731 (13.528)	603 (12.953)	128 (16.639)	0.088
Hyperlipidemia, *n* (%)	2847 (73.165)	2390 (72.883)	457 (74.515)	0.527
DM, *n* (%)				0.001
DM	549 (10.272)	439 (9.314)	110 (14.860)	
IFG	301 (6.592)	250 (6.319)	51 (7.898)	
No	2914 (83.136)	2483 (84.367)	431 (77.242)	
Cancer, *n* (%)	353 (8.687)	274 (7.772)	79 (13.067)	0.006
Anemia, *n* (%)	229 (4.291)	188 (4.127)	41 (5.081)	0.237
Smoking status^∗^, *n* (%)				< 0.001
Current smoker	858 (24.899)	672 (22.443)	186 (36.668)	
Former smoker	1055 (25.944)	884 (25.926)	171 (26.026)	
Never smoker	1851 (49.157)	1616 (51.631)	235 (37.306)	
Alcohol status^#^, *n* (%)				< 0.001
Former drinking	807 (17.940)	647 (16.492)	160 (24.875)	
Heavy drinking	711 (19.968)	602 (19.867)	109 (20.452)	
Mild drinking	1226 (34.589)	1063 (35.593)	163 (29.776)	
Moderate drinking	483 (15.350)	399 (15.245)	84 (15.856)	
Never drinking	537 (12.153)	461 (12.802)	76 (9.041)	
Education, *n* (%)				0.008
Under high school	1096 (18.151)	908 (17.223)	188 (22.594)	
High school or equivalent	920 (26.820)	765 (26.253)	155 (29.537)	
Above high school	1748 (55.029)	1499 (56.523)	249 (47.869)	
NLR	2.249 (0.028)	2.211 (0.023)	2.427 (0.092)	0.025
SII	598.673 (8.557)	585.330 (7.734)	662.603 (23.971)	0.003
WBC (× 10^9^/L)	6.752 (0.046)	6.632 (0.038)	7.331 (0.131)	< 0.001
LYM (× 10^9^/L)	29.571 (0.223)	29.727 (0.211)	28.825 (0.592)	0.137
MON (× 10^9^/L)	8.176 (0.041)	8.219 (0.050)	7.969 (0.089)	0.036
NEU (× 10^9^/L)	58.589 (0.241)	58.389 (0.235)	59.548 (0.641)	0.089
Fasting glucose, mg/dL	101.650 (0.616)	101.170 (0.638)	103.949 (1.861)	0.174
Fasting triglycerides, mg/dL	147.183 (3.022)	143.684 (3.111)	163.946 (7.939)	0.018
RDW (%)	12.640 (0.026)	12.626 (0.026)	12.709 (0.060)	0.163
Albumin (g/L)	42.370 (0.087)	42.500 (0.087)	41.748 (0.164)	< 0.001
BRI	4.960 (0.044)	4.877 (0.046)	5.362 (0.112)	< 0.001
ALT (U/L)	26.618 (0.766)	26.066 (0.463)	29.265 (4.020)	0.439
Serum iron (ug/dL)	93.606 (0.988)	94.568 (1.035)	88.995 (2.014)	0.013
HB (g/dL)	14.629 (0.058)	14.657 (0.060)	14.493 (0.084)	0.040
Waist circumference (cm)	96.959 (0.323)	96.401 (0.338)	99.633 (0.799)	0.001
TyG index	8.704 (0.016)	8.678 (0.018)	8.829 (0.037)	0.001
TyG index group, *n* (%)				0.006
Q1	754 (22.192)	656 (23.222)	98 (17.258)	
Q2	752 (21.128)	643 (21.414)	109 (19.756)	
Q3	752 (19.945)	637 (20.036)	115 (19.509)	
Q4	751 (18.519)	624 (18.427)	127 (18.961)	
Q5	755 (18.216)	612 (16.901)	143 (24.515)	

*Note:* Data were presented as mean and standard errors (SEs) for continuous variables and number and proportions for categorical variables. LYM, lymphocyte; MON, monocytes; NEU, neutrophils; RDW, red cell distribution width; ALT, alanine aminotransferase; HB, hemoglobin; SII, systemic immune inflammation index; CVD, cardiovascular disease.

Abbreviations: BMI, body mass index; BRI, body roundness index; CKD, chronic kidney disease; DM, diabetes mellitus; IFG, impaired fasting glucose; NLR, neutrophil–lymphocyte ratio; TyG, triglyceride–glucose; WBC, white blood cell.

^∗^Smoking status (categorized as never smoker [< 100 cigarettes lifetime], former smoker [> 100 cigarettes lifetime but currently abstinent], or current smoker [> 100 cigarettes lifetime and currently smoking]).

^#^Alcohol status (classified as never [< 12 drinks lifetime], mild [≤ 1 drink/day for women, ≤ 2 drinks/day for men], moderate [2 drinks/day for women, 3 drinks/day for men or binge drinking 2–4 days/month], heavy [≥ 3 drinks/day for women, ≥ 4 drinks/day for men or binge drinking ≥ 5 days/month], or former [≥ 12 drinks/year but abstinent in previous year]).

#### 3.1.2. TyG Index and Chronic Pain Association

Our analysis revealed consistent associations between the TyG index and chronic pain across multiple models. In continuous variable analysis, multivariate‐adjusted odds ratios (ORs) remained significant: Model 1 (OR = 1.40, 95% CI = 1.17–1.67, *p* < 0.001), Model 2 (OR = 1.38, 95% CI = 1.17–1.62, *p* < 0.001), Model 3 (OR = 1.37, 95% CI = 1.06–1.77, *p* = 0.020), and Model 4 (OR = 1.25, 95% CI = 1.04–1.49, *p* = 0.020). When analyzed categorically, participants in the highest TyG quintile (Q5) showed significantly elevated chronic pain risk compared to the reference group (Q1), with adjusted ORs of 1.95 (95% CI = 1.32–2.88, *p* = 0.002) in Model 1, 1.89 (95% CI = 1.32–2.71, *p* = 0.001) in Model 2, 1.84 (95% CI = 1.06–3.19, *p* = 0.040) in Model 3, and 1.58 (95% CI = 1.09–2.31, *p* = 0.020) in Model 4 (Table 2).

**TABLE 2:** Multivariable logistic regression results between TyG and chronic pain.

Variables	Model 1		Model 2		Model 3		Model 4	
	OR (95% CI)	*p* value	OR (95% CI)	*p* value	OR (95% CI)	*p* value	OR (95% CI)	*p* value
Continuous	1.40 (1.17, 1.67)	< 0.001	1.38 (1.17, 1.62)	< 0.001	1.37 (1.06, 1.77)	0.020	1.25 (1.04, 1.49)	0.020
TyG index								
Q1	Ref		Ref		Ref		Ref	
Q2	1.24 (0.87, 1.78)	0.230	1.22 (0.85, 1.75)	0.270	1.25 (0.80, 1.93)	0.250	1.14 (0.80, 1.63)	0.430
Q3	1.31 (0.93, 1.85)	0.120	1.27 (0.91, 1.77)	0.150	1.22 (0.80, 1.86)	0.270	1.14 (0.81, 1.59)	0.430
Q4	1.38 (0.90, 2.14)	0.140	1.32 (0.87, 1.99)	0.180	1.36 (0.77, 2.42)	0.220	1.13 (0.74, 1.72)	0.550
Q5	1.95 (1.32, 2.88)	0.002	1.89 (1.32, 2.71)	0.001	1.84 (1.06, 3.19)	0.040	1.58 (1.09, 2.31)	0.020
P for trend		0.003		0.002		0.030		0.030

*Note:* Model 1: unadjusted. Model 2: adjusted for age, sex, education. Model 3: adjusted for age, education, sex, cardiovascular disease (CVD), hyperlipidemia, albumin, neutrophil–lymphocyte ratio (NLR), serum iron, hemoglobin (HB), smoke status, alcohol status, cancer, anemia, chronic kidney disease (CKD), body mass index (BMI), diabetes mellitus (DM). Model 4: adjusted for age, education, sex, neutrophil–lymphocyte ratio (NLR), smoke status, cancer, chronic kidney disease (CKD), body mass index (BMI), diabetes mellitus (DM).

Abbreviations: CI, confidence intervals; OR, odds ratio; TyG, triglyceride–glucose.

**FIGURE 2:**
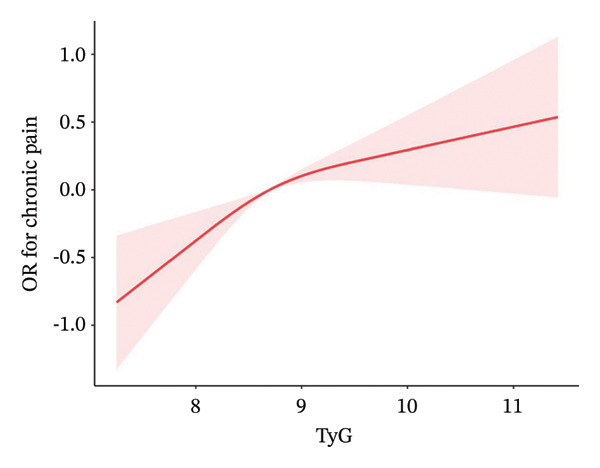
Restricted cubic spline curve for the association between the TyG index and the risk of chronic pain. Red lines represent odds ratios, and pink areas represent 95% confidence intervals. The model was adjusted for age, sex, education, cardiovascular disease, hyperlipidemia, albumin levels, NLR, serum iron, hemoglobin, smoking status, alcohol consumption, cancer, anemia, chronic kidney disease, BMI, and diabetes (*p* < 0.001; NL‐*p* value: 0.098).

RCS analysis with full adjustment (Model 3) demonstrated a significant inverse linear relationship between the TyG index and chronic pain (*p* < 0.001; nonlinear *p* value = 0.098; Figure 2).

**FIGURE 3:**
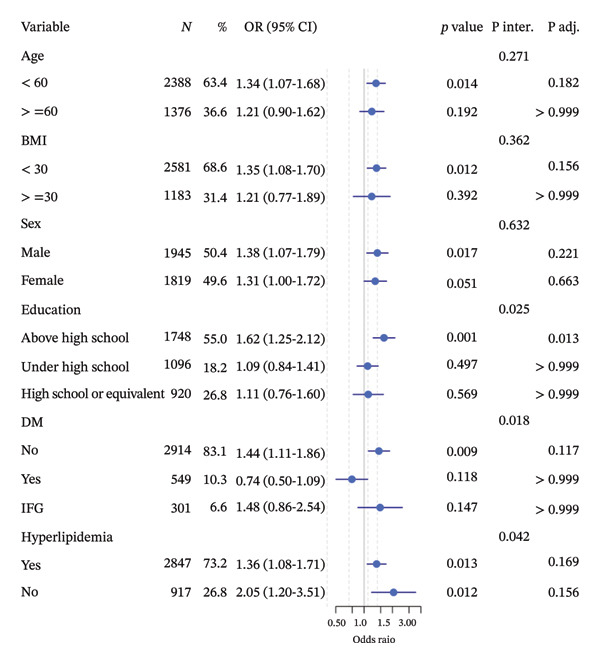
Subgroup analysis of the association between the TyG index and chronic pain. Adjusted for cardiovascular disease (CVD), albumin, neutrophil–lymphocyte ratio (NLR), serum iron, hemoglobin (HB), smoke status, alcohol status, cancer, anemia, and chronic kidney disease (CKD). OR: odds ratio, CI: confidence interval, P inter.: P for interaction, P adj.: P adjusted by Bonferroni.

Subgroup analyses stratified by age, sex, BMI, education level, DM, and hyperlipidemia suggested potential differences in the association between the TyG index and chronic pain across these strata (Figure 3). However, after applying Bonferroni correction for multiple comparisons, only the subgroup with education levels exceeding high school retained statistical significance. Therefore, findings from other subgroups—particularly those showing suggestive but uncorrected associations—should be interpreted with caution and considered exploratory rather than definitive. These observations highlight the need for future studies with larger sample sizes and prespecified subgroup hypotheses to validate potential effect modification.

#### 3.1.3. Mediation Analyses

To investigate potential intermediaries, we assessed WBC count as a mediator, finding it potentially accounted for 12.0% (95% CI: 2.6%–23.1%) of the TyG index’s total effect on chronic pain risk. We also found that WBC potentially accounted for 12.0% (95% CI: 1.0%–23.1%) of the TyG index’s total effect on death in chronic pain patients (Figure 4). However, we found that NLR had no mediating effect on the TyG index’s total effect on chronic pain risk (Figure S1).

FIGURE 4Mediation analyses: A, mediation effects of white blood cell (WBC) count on the TyG index–chronic pain (CP) relationship; B, mediation effects of white blood cell (WBC) count on TyG index–death relationship in chronic pain patients.

**Figure:**
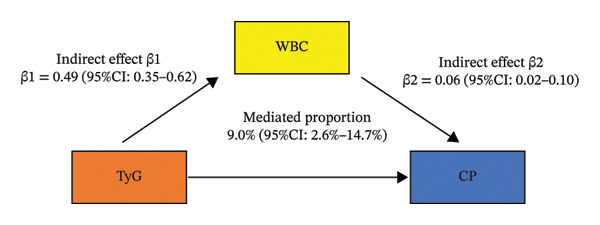
(a)

**Figure:**
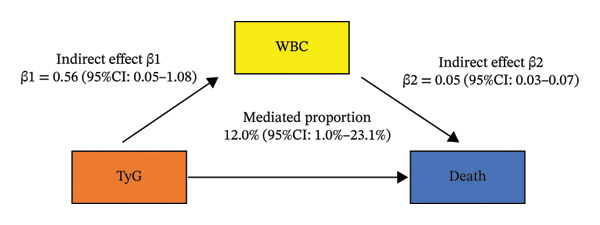
(b)

### 3.2. Cohort Study Analysis

#### 3.2.1. Basic Characteristics

The mortality analysis included 591 chronic pain patients (43.7% male, *n* = 258; 56.3% female, *n* = 333), with 32.1% (*n* = 190) mortality during follow‐up. The median follow‐up period was 195.0 (165.5211.0) months. Approximately 32.1% (*n* = 190) of the participants in our study died. The participants who died had a higher incidence of CVD, CKD, hyperlipidemia, DM, and cancer. Additional demographic and clinical characteristics are detailed in Supporting Table S1.

#### 3.2.2. Association Between TyG Index and All‐Cause Mortality in Chronic Pain Patients

**FIGURE 5:**
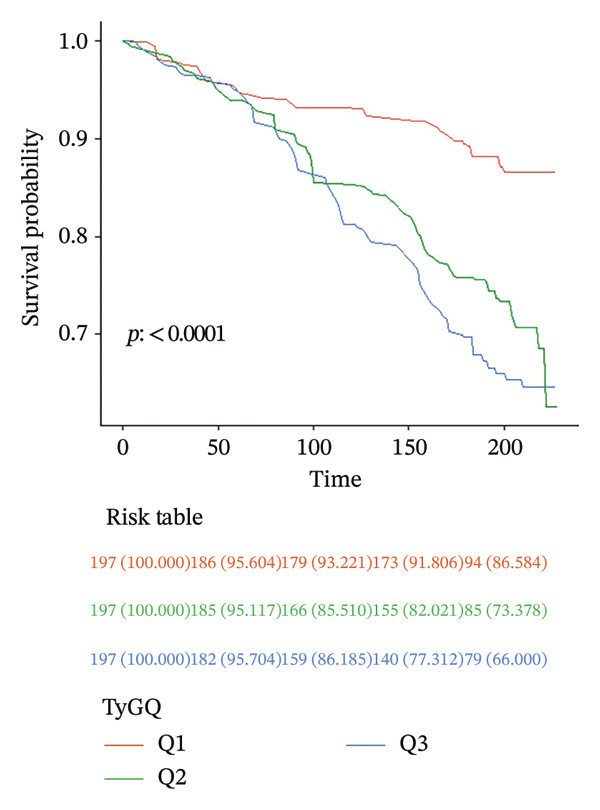
Kaplan–Meier survival curves for all‐cause mortality in participants with chronic pain by TyG index.

Analysis of TyG index–mortality associations demonstrated consistent dose‐dependent patterns in all models. Continuous TyG elevation showed progressively attenuated but persistent mortality risk: Model 1 HR = 1.65 (95% CI: 1.28–2.12, *p* < 0.001), Model 2 adjusted = 1.33 (95% CI: 1.02–1.73, *p* = 0.034), Model 3 adjusted = 1.30 (95% CI: 1.01–1.68, *p* = 0.043), and Model 4 adjusted = 1.26 (95% CI: 1.04–1.53, *p* = 0.018). Tertile‐based comparisons revealed particularly elevated risk in T3 versus T1 (Model 4 adjusted HR = 2.05, 95% CI: 1.46–2.89, *p* < 0.001) (Table 3). Survival curves confirmed progressively worse outcomes with higher TyG tertiles (log‐rank *p* < 0.001; Figure 5). Schoenfeld residuals showed there were no significant violations in Model 4 (global *p* > 0.05).

**TABLE 3:** Multivariable Cox regression results between TyG and all‐cause mortality in participants with chronic pain.

Variables	Model 1		Model 2		Model 3		Model 4	
	HR (95% CI)	*p* value	HR (95% CI)	*p* value	HR (95% CI)	*p* value	HR (95% CI)	*p* value
Continuous	1.65 (1.28,2.12)	< 0.001	1.33 (1.02,1.73)	0.034	1.30 (1.01,1.68)	0.043	1.26 (1.04,1.53)	0.018
TyG index								
T1	Ref		Ref		Ref		Ref	
T2	2.42 (1.45,4.04)	< 0.001	1.42 (0.84,2.39)	0.188	1.51 (0.89,2.55)	0.124	1.44 (0.87,2.39)	0.160
T3	2.97 (1.84,4.79)	< 0.001	1.85 (1.18,2.88)	0.007	2.34 (1.53,3.57)	< 0.001	2.05 (1.46,2.89)	< 0.001
P for trend		< 0.001		0.005		< 0.001		< 0.001

*Note:* Model 1: unadjusted. Model 2: adjusted for age, sex, education. Model 3: adjusted for age, education, sex, cardiovascular disease (CVD), hyperlipidemia, albumin, neutrophil–lymphocyte ratio (NLR), serum iron, hemoglobin (HB), smoke status, alcohol status, cancer, chronic kidney disease (CKD), body mass index (BMI), diabetes mellitus (DM). Model 4: adjusted for age, education, sex, cardiovascular disease (CVD), albumin, hemoglobin (HB), neutrophil–lymphocyte ratio (NLR), alcohol status, chronic kidney disease (CKD), body mass index (BMI), diabetes mellitus (DM).

Abbreviations: CI, confidence intervals; HR, hazard ratio; TyG, triglyceride‐glucose.

### 3.3. Sensitivity Analysis

We performed a sensitivity analysis using MICE for covariates with missing values. In the cross‐sectional analysis, a total of 4463 participants were included. Comparison of baseline characteristics between the imputed and original groups showed consistent trends (Table S4A). Logistic regression revealed that participants in the highest TyG quintile (Q5) had a significantly increased risk of chronic pain compared to those in the lowest quintile (Q1), with adjusted ORs of 1.97 (95% CI: 1.34–2.91, *p* = 0.001) in Model 1, 1.91 (95% CI: 1.34–2.74, *p* = 0.001) in Model 2, 1.86 (95% CI: 1.07–3.22, *p* = 0.030) in Model 3, and 1.58 (95% CI: 1.09–2.31, *p* = 0.020) in Model 4 (Table S4B).

In the cohort analysis, 696 patients with chronic pain were included, of whom 32.8% (*n* = 228) died during follow‐up. Cox regression indicated a particularly elevated mortality risk in the highest TyG tertile (T3) compared to the lowest (T1), with a Model 4 adjusted HR of 1.95 (95% CI: 1.4–2.69, *p* < 0.001) (Table S4C).

## 4. Discussion

Our findings demonstrated that elevated TyG index levels remained significantly associated with chronic pain prevalence even after full covariate adjustment. Furthermore, survival analyses revealed a clear mortality gradient, with the highest TyG tertiles showing progressively worse survival outcomes in chronic pain patients. Notably, mediation modeling indicated that systemic inflammation (measured by WBC) potentially explained 7.6% (2.6%–14.7%) of this metabolic–pain association. Moreover, in chronic pain patients, inflammation (measured by WBC) potentially mediated 12.0% (95% CI: 1.0%–23.1%) of the observed metabolic–mortality relationship.

Growing evidence underscored the critical involvement of metabolic dysregulation in pain development [20, 21]. Among various metabolic indicators, the TyG index has gained recognition as a clinically relevant metabolic biomarker for pain‐related conditions [22]. Supporting evidence came from a large‐scale NHANES study (2007–2016), which established a significant association between the TyG index and chest pain in individuals with dysglycemia [22]. Our study expanded upon these observations, demonstrating that higher TyG index values were strongly associated with an increased likelihood of chronic pain. Importantly, this relationship remained significant and even strengthened after rigorous adjustment for confounding variables.

While the precise biological mechanisms linking the TyG index to chronic pain pathogenesis require further investigation, our study provided potential mechanistic insights by demonstrating that WBC count served as a significant mediator in the IR–chronic pain pathway. This finding offered a new theoretical framework for understanding the pathophysiological mechanisms connecting IR with chronic pain development.

Prior research has consistently demonstrated a link between IR and sustained chronic inflammation [24, 25]. In clinical practice, total WBC count serves as a reliable biomarker for assessing inflammation and metabolism‐related disorders [25]. Evidence suggested that individuals developing IR and glucose metabolism dysfunction in early life tended to demonstrate elevated WBC counts [27]. Conversely, research has shown that heightened WBC levels could contribute to reduced insulin sensitivity, suggesting that immune system activation might predispose individuals to IR development [28]. This reciprocal relationship between WBC‐mediated inflammation and IR created a complex pathophysiological cycle: IR promoted chronic inflammation through the release of various inflammatory mediators, while persistent inflammatory states further aggravated IR.

The pivotal role of inflammation in chronic pain pathogenesis has been substantiated by multiple lines of evidence [29]. Clinical research by Shu et al. demonstrated a significant association between elevated neutrophil‐to‐lymphocyte ratio (NLR ≥ 5) and increased risk of chronic postsurgical pain following abdominal procedures [30]. Population‐based evidence from the UK Biobank revealed a significant association between C‐reactive protein (CRP) levels and regional chronic pain patterns [31]. Furthermore, genetic evidence from a two‐sample Mendelian randomization analysis suggested that IL‐6 signaling downregulation, as indicated by CRP levels, could potentially confer protection against chronic back and knee pain development [31]. These converging findings established a robust association between inflammatory processes and chronic pain development. The underlying mechanisms involved inflammatory‐mediated release of various signaling molecules, including proinflammatory cytokines, chemokines, and adenosine triphosphate. These mediators interacted with specific receptors on peripheral nociceptive neurons, leading to altered neuronal sensitivity and excitability that perpetuated chronic pain states [33]. However, WBC included diverse cell types with differing roles, such as proinflammatory NEU and regulatory LYMs. Future studies should use more specific markers—like the neutrophil‐to‐lymphocyte ratio or individual cytokines—to clarify how distinct immune cells contribute to pain in metabolic disorders.

The TyG index has proven to be a reliable predictor of mortality risk in various clinical settings. Data from the MIMIC‐IV 3.0 database revealed that higher TyG levels were persistently associated with greater mortality risk over time, particularly among critically ill ischemic stroke patients in the top TyG quartile [34]. Among individuals with diabetes, Feng et al. identified distinct mortality patterns: a J‐shaped relationship with all‐cause mortality (threshold: 9.32) and a reversed L‐shaped association with cardiovascular mortality (threshold: 9.37) [35]. Similarly, NHANES analyses identified an L‐shaped mortality pattern in older hypertensive adults [36]. Our study complements this evidence by showing that chronic pain patients with elevated TyG indices—especially those in the third tertile—face heightened all‐cause mortality risk. Together, these findings highlight the TyG index’s potential as a prognostic tool for mortality risk assessment in chronic pain populations, warranting further clinical exploration.

At the same time, the modest mediating effect of WBC suggested that systemic inflammation potentially explained part of the association between the TyG index and mortality in patients with chronic pain. This limited mediation might be attributed to several factors. First, WBC was a nonspecific inflammatory marker that could reflect a range of conditions beyond metabolic inflammation, including acute infections or subclinical comorbidities. Second, residual confounding from unmeasured variables—such as frailty, subclinical vascular disease, or psychosocial stress—could concurrently influence both inflammation levels and mortality risk. Therefore, while our findings supported the presence of an inflammatory–metabolic pathway potentially contributing to mortality in this population, they should be regarded as preliminary. Future longitudinal studies incorporating more specific immune biomarkers are needed to clarify the causal mechanisms underlying these relationships.

Our study possessed several notable strengths, particularly the utilization of a large, nationally representative sample from the US population, which enhanced the generalizability and statistical power of our findings. However, several limitations warranted consideration. First, despite conducting multiple imputation for covariates, the high exclusion rate (∼64%) from listwise deletion remains a concern and may limit generalizability. Although baseline characteristics were comparable between those with and without missing TyG data—suggesting data were likely missing completely at random—our findings should be interpreted within the context of the complete‐case cohort. Second, the cross‐sectional design precluded the establishment of causal relationships, necessitating future investigations through prospective studies or Mendelian randomization studies to establish causality and the potential impact on chronic pain incidence and mortality outcomes. Third, this study included multiple subgroup and sensitivity analyses, which might increase the risk of false‐positive findings (Type I error). While we applied Bonferroni correction—a conservative method—it might not fully address all multiple comparisons. Therefore, subgroup results, particularly those with borderline significance after correction, should be considered exploratory and confirmed in future studies. Fourth, chronic pain assessment in NHANES relied on self‐reported duration (susceptible to recall bias) and lacked granularity on site, severity, or etiology. This broad definition might dilute subtype‐specific associations. Future studies with objective measures and detailed phenotyping are needed. Finally, while mediation analysis allowed for the statistical inference of potential mechanistic relationships, it was important to note that cross‐sectional data could not establish the temporal sequence among exposure, mediator, and outcome. Consequently, the identified relationship of “IR–inflammation–chronic pain” should be regarded as a biologically plausible hypothesis requiring longitudinal validation, rather than as definitive causal evidence.

In summary, this study established a robust and independent link between the elevated TyG index and chronic pain, which persisted after comprehensive adjustment for potential confounders. Besides, higher TyG levels correlated with progressively poorer survival among individuals with chronic pain. Importantly, systemic inflammation—quantified by WBC count—potentially mediated both the metabolic–pain relationship and the adverse impact of the TyG index on mortality in chronic pain patients. These findings suggested that inflammation partially underlined the detrimental interplay between IR, chronic pain, and long‐term survival, highlighting potential relationships for integrated risk stratification and targeted interventions.

## Author Contributions

Di Zhou: conceptualization, data curation, investigation, methodology, resources, supervision, validation, visualization, and writing–original draft. Fan Jin: conceptualization, data curation, formal analysis, investigation, methodology, resources, supervision, and validation. Ling Zhang: conceptualization, data curation, investigation, methodology, validation, and visualization. Jin Chen: conceptualization, data curation, investigation, methodology, validation, and visualization. Lv Tian: conceptualization, data curation, formal analysis, investigation, methodology, resources, supervision, and writing–review and editing. Jie Li: conceptualization, formal analysis, investigation, methodology, resources, supervision, validation, visualization, and writing–review and editing.

## Funding

No funding was received for this research.

## Ethics Statement

The National Health and Nutrition Examination Survey (NHANES) has been approved by the National Center for Health Statistics Ethics Review Board (No. Protocol#98‐12), and all participants provided informed written consent at enrollment.

## Conflicts of Interest

The authors declare no conflicts of interest.

## Supporting Information

Additional supporting information can be found online in the Supporting Information section.

## Supporting information


**Supporting Information 1** Table S1: Baseline characteristics of study participants based on chronic pain. This table included demographic and clinical characteristics of study participants with chronic pain who were grouped according to whether they died.

**Supporting Information 2** Table S2: Baseline characteristics of participants with or without TyG data.

**Supporting Information 3** Table S3A: Collinearity analysis results of different variables in logistic regression analyses; Table S3B Collinearity analysis results of different variables in Cox regression analyses.

**Supporting Information 4** Table S4A: Baseline characteristics of participants with or without chronic pain after multiple imputation; Table S4B: Multivariable logistic regression results between TyG and chronic pain after multiple imputation; TableS4C: Multivariable Cox regression results between TyG and all‐cause mortality in participants with chronic pain after multiple imputation.

**Supporting Information 5** Figure S1: Mediation analyses: mediation effects of the neutrophil–lymphocyte ratio (NLR) on the TyG index–chronic pain (CP) relationship.

## Data Availability

All data are available upon request from the corresponding author.
