# Supplementary material for: The Mediating Role of WBC in the Relationship Between Triglyceride–Glucose Index and Chronic Pain: Evidence From NHANES 2001–2004 Data
Source: Pain Res Manag. 2026 Apr 21;2026:3793191. doi: 10.1155/prm/3793191 (PMC13096791; doi:10.1155/prm/3793191)
Supplement: Supplementary file 1 — Supporting Information 1 Table S1: Baseline characteristics of study participants based on chronic pain. This table included demographic and clinical characteristics of study participants with chronic pain who were grouped according to whether they died. [file PRM-2026-3793191-s001.docx]

Table S1 Baseline characteristics of study participants based on the chronic pain

| Variables | Overall (n=591) | No death  (n=401) | Death  (n=190) | P-value |
| --- | --- | --- | --- | --- |
| Age, years | 48.507(0.745) | 44.678(0.829) | 60.109(1.334) | <0.001 |
| Sex, n (%) |  |  |  | 0.051 |
| Female | 333(56.128) | 240(59.062) | 93(47.237) |  |
| Male | 258(43.872) | 161(40.938) | 97(52.763) |  |
| BMI, kg/m^2^ | 29.225(0.311) | 28.995(0.302) | 29.919(0.705) | 0.206 |
| CVD, n (%) | 119(16.216) | 49(9.418) | 70(36.812) | <0.001 |
| CKD, n (%) | 127(16.551) | 43(9.594) | 84(37.707) | <0.001 |
| Hyperlipidemia, n (%) | 457(74.564) | 299(71.851) | 158(82.785) | 0.012 |
| DM, n (%) |  |  |  | <0.001 |
| DM | 109(14.804) | 43(9.279) | 66(31.546) |  |
| IFG | 51(7.903) | 32(7.456) | 19(9.255) |  |
| No | 431(77.293) | 326(83.265) | 105(59.199) |  |
| Cancer, n (%) | 79(13.075) | 32(7.445) | 47(30.137) | <0.001 |
| Anemia, n (%) | 41(5.084) | 18(4.201) | 23(7.758) | 0.121 |
| Smoking status^&^, n (%) |  |  |  | 0.047 |
| Current smoker | 186(36.692) | 138(38.398) | 48(31.524) |  |
| Former smoker | 170(25.977) | 98(22.755) | 72(35.741) |  |
| Never smoker | 235(37.331) | 165(38.848) | 70(32.735) |  |
| Alcohol status^#^, n (%) |  |  |  | 0.004 |
| Former drinking | 160(24.892) | 92(21.122) | 68(36.314) |  |
| Heavy drinking | 109(20.466) | 85(22.208) | 24(15.187) |  |
| Mild drinking | 162(29.730) | 114(30.439) | 48(27.582) |  |
| Moderate drinking | 84(15.866) | 66(17.657) | 18(10.438) |  |
| Never drinking | 76(9.047) | 44(8.574) | 32(10.479) |  |
| Education, n (%) |  |  |  | <0.001 |
| Under high school | 188(22.609) | 102(17.087) | 86(39.339) |  |
| High school or equivalent | 248(47.834) | 183(50.792) | 65(38.874) |  |
| Above high school | 155(29.557) | 116(32.121) | 39(21.788) |  |
| NLR | 2.427(0.093) | 2.342(0.107) | 2.685(0.152) | 0.054 |
| SII | 662.705(23.986) | 633.155(27.873) | 752.240(55.593) | 0.072 |
| WBC, ×10^9^/L | 7.331(0.132) | 7.147(0.120) | 7.891(0.373) | 0.065 |
| LYM, ×10^9^/L | 28.827(0.592) | 29.104(0.605) | 27.988(0.969) | 0.231 |
| MON, ×10^9^/L | 7.968(0.089) | 7.937(0.093) | 8.059(0.256) | 0.671 |
| NEU, ×10^9^/L | 59.552(0.641) | 59.381(0.699) | 60.068(0.880) | 0.444 |
| Fasting glucose, mg/dL | 103.937(1.862) | 99.998(1.261) | 115.874(4.433) | < 0.001 |
| Fasting triglycerides, mg/dL | 163.992(7.947) | 159.543(10.089) | 177.472(7.406) | 0.157 |
| RWD, % | 12.708(0.059) | 12.565(0.067) | 13.144(0.135) | < 0.001 |
| Albumin, g/L | 41.748(0.164) | 41.979(0.166) | 41.050(0.320) | 0.006 |
| BRI | 5.362(0.112) | 5.138(0.115) | 6.041(0.250) | 0.002 |
| ALT, U/L | 29.265(4.022) | 24.986(0.847) | 42.230(16.354) | 0.303 |
| Serum iron, ug/dl | 89.017(2.015) | 90.572(2.258) | 84.303(3.106) | 0.075 |
| HB, g/dl | 14.495(0.084) | 14.550(0.111) | 14.326(0.122) | 0.228 |
| Waist circumference, cm | 99.642(0.800) | 98.237(0.944) | 103.899(1.442) | 0.002 |
| TyG index | 8.829(0.037) | 8.761(0.041) | 9.035(0.058) | < 0.001 |
| Time (months) | 181.418(2.312) | 203.675(1.259) | 113.981(6.834) | < 0.0001 |

Note: Data were presented as mean and standard errors (SE) for continuous variables, number and proportions for categorical variables. BMI, body mass index; CVD, cardiovascular disease; CKD, chronic kidney disease; DM, diabetes mellitus; IFG, impaired fasting glucose; NLR, neutrophil–lymphocyte ratio; SII, systemic immune inflammation index; WBC, white blood cell; LYM, lymphocyte; MON, monocytes; NEU, neutrophils; RWD, red cell distribution width; BRI, body roundness index; ALT, alanine aminotransferase; HB, hemoglobin; TyG, triglyceride-glucose.

^&^ Smoking status (categorized as: **never smoker** [<100 cigarettes lifetime], **former smoker** [>100 cigarettes lifetime but currently abstinent], or **current smoker** [>100 cigarettes lifetime and currently smoking])

^#^ Alcohol status (classified as: **never** [<12 drinks lifetime], **mild** [≤1 drink/day for women, ≤2 drinks/day for men], **moderate** [2 drinks/day for women, 3 drinks/day for men or binge drinking 2-4 days/month], **heavy** [≥3 drinks/day for women, ≥4 drinks/day for men or binge drinking ≥5 days/month], or **former** [≥12 drinks/year but abstinent in previous year])
