# Supplementary material for: The Mediating Role of WBC in the Relationship Between Triglyceride–Glucose Index and Chronic Pain: Evidence From NHANES 2001–2004 Data
Source: Pain Res Manag. 2026 Apr 21;2026:3793191. doi: 10.1155/prm/3793191 (PMC13096791; doi:10.1155/prm/3793191)
Supplement: Supplementary file 2 — Supporting Information 2 Table S2: Baseline characteristics of participants with or without TyG data. [file PRM-2026-3793191-s002.docx]

Table S2：Baseline characteristics of participants with or without TyG data

| Variables | Overall (n=10442) | TyG data (n=4463) | Missing TyG data  (n=5979) | p-value |
| --- | --- | --- | --- | --- |
| Age, years | 50.40 ± 19.60 | 49.88 ± 19.14 | 50.78 ± 19.92 | 0.02 |
| Sex, n (%) |  |  |  | 0.21 |
| Female | 5490(52.58) | 2314(51.85) | 3176(53.12) |  |
| Male | 4952(47.42) | 2149(48.15) | 2803(46.88) |  |
| BMI, kg/m^2^ | 28.30 ± 6.24 | 28.36 ± 6.19 | 28.25 ± 6.28 | 0.40 |
| CVD, n (%) | 1324(12.68) | 513(11.49) | 811(13.57) | <0.01 |
| CKD, n (%) | 1829(20.05) | 874(19.82) | 955(20.28) | 0.60 |
| Hyperlipidemia, n (%) | 6805(69.73) | 3372(75.55) | 3433(64.82) | <0.001 |
| DM, n (%) |  |  |  | <0.001 |
| DM | 1380(13.95) | 634(15.02) | 746(13.15) |  |
| IFG | 341(3.45) | 341(8.08) | 0(0) |  |
| No | 8171(82.60) | 3246(76.90) | 4925(86.85) |  |
| Cancer, n (%) | 998(9.57) | 408(9.15) | 590(9.89) | 0.22 |
| Anemia, n (%) | 685(7.37) | 311(6.97) | 374(7.73) | 0.16 |
| Smoking status^&^, n (%) |  |  |  | 0.36 |
| Current smoker | 2301(22.07) | 981(22.01) | 1320(22.12) |  |
| Former smoker | 2785(26.71) | 1222(27.41) | 1563(26.19) |  |
| Never smoker | 5339(51.21) | 2255(50.58) | 3084(51.68) |  |
| Alcohol status^#^, n (%) |  |  |  | 0.20 |
| Former drinking | 1894(21.24) | 922(22.13) | 972(20.46) |  |
| Heavy drinking | 1656(18.57) | 769(18.46) | 887(18.67) |  |
| Mild drinking | 2833(31.77) | 1323(31.76) | 1510(31.79) |  |
| Moderate drinking | 1183(13.27) | 523(12.55) | 660(13.89) |  |
| Never drinking | 1350(15.14) | 629(15.10) | 721(15.18) |  |
| Education, n (%) |  |  |  | 0.33 |
| 9-11th Grade | 1629(15.65) | 680(15.26) | 949(15.94) |  |
| College Graduate or above | 1994(19.16) | 841(18.87) | 1153(19.37) |  |
| High School Grad/GED or Equivalent | 2532(24.33) | 1065(23.90) | 1467(24.65) |  |
| Less Than 9th Grade | 1521(14.61) | 657(14.74) | 864(14.52) |  |
| Some College or AA degree | 2732(26.25) | 1213(27.22) | 1519(25.52) |  |
| NLR | 2.32 ± 1.27 | 2.35 ± 1.27 | 2.29 ± 1.28 | 0.03 |
| SII | 616.80 ± 418.95 | 617.85 ± 425.94 | 615.82 ± 412.42 | 0.82 |
| WBC, ×10^9^/L | 7.31 ± 2.51 | 6.91 ± 2.35 | 7.68 ± 2.61 | <0.001 |
| LYM, ×10^9^/L | 29.56 ± 8.69 | 29.20 ± 8.70 | 29.90 ± 8.67 | <0.001 |
| MON, ×10^9^/L | 7.89 ± 2.27 | 8.10 ± 2.31 | 7.69 ± 2.22 | <0.001 |
| NEU, ×10^9^/L | 59.13 ± 9.68 | 59.15 ± 9.71 | 59.11 ± 9.66 | 0.84 |
| Fasting glucose, mg/dL | 104.73 ± 35.72 | 104.78 ± 35.75 | 100.82 ± 33.44 | 0.37 |
| Fasting triglycerides, mg/dL | 153.08 ± 145.53 | 153.07 ± 145.60 | 161.20 ± 66.04 | 0.80 |
| RWD, % | 12.82 ± 1.22 | 12.84 ± 1.26 | 12.81 ± 1.18 | 0.32 |
| Albumin, g/L | 41.96 ± 3.70 | 41.60 ± 3.63 | 42.31 ± 3.73 | <0.001 |
| BRI | 5.19 ± 2.06 | 5.19 ± 2.05 | 5.13 ± 2.13 | 0.68 |
| ALT, U/L | 25.20 ± 32.87 | 25.69 ± 44.14 | 24.74 ± 15.96 | 0.18 |
| Serum iron, ug/dl | 86.50 ± 36.58 | 90.68 ± 37.46 | 82.54 ± 35.27 | <0.001 |
| HB, g/dl | 14.26 ± 1.56 | 14.33 ± 1.59 | 14.20 ± 1.54 | <0.001 |
| Waist circumference, cm | 97.45 ± 15.07 | 97.60 ± 14.87 | 97.32 ± 15.25 | 0.37 |

Note: Data were presented as mean and Standard deviation (SD) for continuous variables, number and proportions for categorical variables. BMI, body mass index; CVD, cardiovascular disease; CKD, chronic kidney disease; DM, diabetes mellitus; IFG, impaired fasting glucose; NLR, neutrophil–lymphocyte ratio; SII, systemic immune inflammation index; WBC, white blood cell; LYM, lymphocyte; MON, monocytes; NEU, neutrophils; RWD, red cell distribution width; BRI, body roundness index; ALT, alanine aminotransferase; HB, hemoglobin; TyG, triglyceride-glucose.

^&^ Smoking status (categorized as: never smoker [<100 cigarettes lifetime], former smoker [>100 cigarettes lifetime but currently abstinent], or current smoker [>100 cigarettes lifetime and currently smoking])

^#^ Alcohol status (classified as: never [<12 drinks lifetime], mild [≤1 drink/day for women, ≤2 drinks/day for men], moderate [2 drinks/day for women, 3 drinks/day for men or binge drinking 2-4 days/month], heavy [≥3 drinks/day for women, ≥4 drinks/day for men or binge drinking ≥5 days/month], or former [≥12 drinks/year but abstinent in previous year])
