# Supplementary material for: The Mediating Role of WBC in the Relationship Between Triglyceride–Glucose Index and Chronic Pain: Evidence From NHANES 2001–2004 Data
Source: Pain Res Manag. 2026 Apr 21;2026:3793191. doi: 10.1155/prm/3793191 (PMC13096791; doi:10.1155/prm/3793191)
Supplement: Supplementary file 3 — Supporting Information 3 Table S3A: Collinearity analysis results of different variables in logistic regression analyses; Table S3B Collinearity analysis results of different variables in Cox regression analyses. [file PRM-2026-3793191-s003.docx]

Table S3A Collinearity analysis results of different variables in logistic regression analyses

| Variables | VIF |
| --- | --- |
| TyG | 3.75 |
| Age | 9.49 |
| Education |  |
| college | 13.73 |
| high school | 9.85 |
| Male | 12.29 |
| CVD | 10.94 |
| Hyperlipidemia | 10.02 |
| albumin | 16.87 |
| NLR | 6.85 |
| serum iron | 14.54 |
| HB | 24.12 |
| Smoke |  |
| never | 9.22 |
| now | 6.79 |
| Alcohol status |  |
| Heavy drinking | 10.17 |
| Mild drinking | 10.39 |
| Moderate drinking | 12.35 |
| Never drinking | 10.35 |
| cancer | 4.30 |
| Anemia | 11.05 |
| CKD | 4.71 |
| DM |  |
| IFG | 6.93 |
| no | 10.97 |
| BMI | 6.16 |

Note: VIF, Variance Inflation Factor; TyG, triglyceride-glucose; CVD, cardiovascular disease; NLR, neutrophil–lymphocyte ratio; HB, hemoglobin; CKD, chronic kidney disease; DM, diabetes mellitus; IFG, impaired fasting glucose; BMI, body mass index.

Moderate collinearity: 5<VIF≤10

Heavy collinearity: VIF>10

Table S3B Collinearity analysis results of different variables in Cox regression analyses

| Variables | VIF |
| --- | --- |
| TyG | 9.18 |
| Age | 27.11 |
| Education |  |
| college | 7.20 |
| high school | 13.30 |
| Male | 6.62 |
| CVD | 6.51 |
| Hyperlipidemia | 20.16 |
| albumin | 8.52 |
| NLR | 8.40 |
| serum iron | 12.83 |
| HB | 6.36 |
| Smoke |  |
| never | 17.28 |
| now | 18.86 |
| Alcohol status |  |
| Heavy drinking | 11.93 |
| Mild drinking | 13.57 |
| Moderate drinking | 13.30 |
| Never drinking | 7.42 |
| cancer | 44.90 |
| CKD | 4.09 |
| DM |  |
| IFG | 4.96 |
| no | 6.71 |
| BMI | 8.37 |

Note: VIF, Variance Inflation Factor; TyG, triglyceride-glucose; CVD, cardiovascular disease; NLR, neutrophil–lymphocyte ratio; HB, hemoglobin; CKD, chronic kidney disease; DM, diabetes mellitus; IFG, impaired fasting glucose; BMI, body mass index.

Moderate collinearity: 5<VIF≤10

Heavy collinearity: VIF>10
