# Supplementary material for: The Mediating Role of WBC in the Relationship Between Triglyceride–Glucose Index and Chronic Pain: Evidence From NHANES 2001–2004 Data
Source: Pain Res Manag. 2026 Apr 21;2026:3793191. doi: 10.1155/prm/3793191 (PMC13096791; doi:10.1155/prm/3793191)
Supplement: Supplementary file 4 — Supporting Information 4 Table S4A: Baseline characteristics of participants with or without chronic pain after multiple imputation; Table S4B: Multivariable logistic regression results between TyG and chronic pain after multiple imputation; TableS4C: Multivariable Cox regression results between TyG and all‐cause mortality in participants with chronic pain after multiple imputation. [file PRM-2026-3793191-s004.docx]

TableS4A：Baseline characteristics of participants with or without chronic pain after multiple imputation

| Variables | Overall (n=4463) | No pain  (n=3767) | Chronic pain  (n=696) | p-value |
| --- | --- | --- | --- | --- |
| Age, years | 46.066(0.533) | 45.506(0.608) | 48.721(0.788) | 0.002 |
| Sex, n (%) |  |  |  | 0.001 |
| Female | 2314(51.031) | 1906(49.560) | 408(58.004) |  |
| Male | 2149(48.969) | 1861(50.440) | 288(41.996) |  |
| BMI, kg/m^2^ | 28.152(0.118) | 27.927(0.136) | 29.221(0.247) | < 0.001 |
| CVD, n (%) | 513(8.253) | 370(6.560) | 143(16.278) | <0.001 |
| Hyperlipidemia, n (%) | 3372(72.842) | 2833(72.486) | 539(74.530) | 0.371 |
| DM, n (%) |  |  |  | 0.002 |
| DM | 634(10.403) | 504(9.661) | 130(14.963) |  |
| IFG | 341(6.752) | 282(6.603) | 59(8.148) |  |
| No | 3246(81.066) | 2761(83.736) | 485(76.889) |  |
| Cancer, n (%) | 408(8.545) | 311(7.464) | 97(13.783) | 0.001 |
| Anemia, n (%) | 311(4.763) | 249(4.453) | 62(6.241) | 0.052 |
| Smoking status^&^, n (%) |  |  |  | < 0.001 |
| Current smoker | 1222(25.625) | 1017(25.422) | 205(26.718) |  |
| Former smoker | 2255(49.650) | 1980(52.452) | 275(36.627) |  |
| Never smoker | 981(24.638) | 765(22.126) | 216(36.655) |  |
| Alcohol status^#^, n (%) |  |  |  | < 0.001 |
| Former drinking | 922(17.289) | 735(16.937) | 187(25.312) |  |
| Heavy drinking | 769(18.552) | 653(19.659) | 116(20.208) |  |
| Mild drinking | 1323(31.994) | 1147(35.187) | 176(28.831) |  |
| Moderate drinking | 523(14.115) | 434(14.992) | 89(15.213) |  |
| Never drinking | 629(11.960) | 536(13.226) | 93(10.436) |  |
| Education, n (%) |  |  |  | 0.010 |
| Under high school | 1337(19.017) | 1114(18.307) | 223(22.536) |  |
| High school or equivalent | 2054(54.729) | 1760(56.184) | 294(48.314) |  |
| Above high school | 1065(26.105) | 886(25.509) | 179(29.150) |  |
| NLR | 2.289(0.028) | 2.253(0.026) | 2.461(0.077) | 0.012 |
| WBC, ×10^9^/L | 6.824(0.050) | 6.705(0.042) | 7.385(0.120) | <0.001 |
| LYM, ×10^9^/L | 29.320(0.221) | 29.481(0.227) | 28.554(0.491) | 0.081 |
| MON, ×10^9^/L | 8.121(0.037) | 8.160(0.044) | 7.935(0.096) | 0.060 |
| NEU, ×10^9^/L | 58.939(0.245) | 58.737(0.252) | 59.896(0.551) | 0.055 |
| RWD, % | 12.666(0.025) | 12.653(0.024) | 12.730(0.061) | 0.185 |
| Albumin, g/L | 42.178(0.079) | 42.322(0.078) | 41.497(0.181) | < 0.001 |
| ALT, U/L | 26.263(0.669) | 25.750(0.406) | 28.698(3.548) | 0.421 |
| Serum iron, ug/dl | 93.036(0.874) | 93.948(0.965) | 88.714(1.805) | 0.015 |
| HB, g/dl | 14.563(0.053) | 14.594(0.056) | 14.418(0.076) | 0.021 |
| Waist circumference, cm | 96.970(0.298) | 96.393(0.319) | 99.704(0.653) | < 0.001 |
| TyG index | 8.713(0.016) | 8.685(0.017) | 8.848(0.041) | < 0.001 |
| TyG index group, n (%) |  |  |  | 0.001 |
| Q1 | 892(22.404) | 778(23.368) | 114(17.836) | |
| Q2 | 893(21.082) | 770(21.582) | 123(18.712) | |
| Q3 | 895(19.808) | 759(19.934) | 136(19.210) | |
| Q4 | 891(18.414) | 746(18.314) | 145(18.887) | |
| Q5 | 892(18.292) | 714(16.802) | 178(25.355) | |

Note: Data were presented as mean and standard errors (SE) for continuous variables, number and proportions for categorical variables. BMI, body mass index; CVD, cardiovascular disease; DM, diabetes mellitus; IFG, impaired fasting glucose; NLR, neutrophil–lymphocyte ratio; WBC, white blood cell; LYM, lymphocyte; MON, monocytes; NEU, neutrophils; RWD, red cell distribution width; ALT, alanine aminotransferase; HB, hemoglobin; TyG, triglyceride-glucose.

^&^ Smoking status (categorized as: **never smoker** [<100 cigarettes lifetime], **former smoker** [>100 cigarettes lifetime but currently abstinent], or **current smoker** [>100 cigarettes lifetime and currently smoking])

^#^ Alcohol status (classified as: **never** [<12 drinks lifetime], **mild** [≤1 drink/day for women, ≤2 drinks/day for men], **moderate** [2 drinks/day for women, 3 drinks/day for men or binge drinking 2-4 days/month], **heavy** [≥3 drinks/day for women, ≥4 drinks/day for men or binge drinking ≥5 days/month], or **former** [≥12 drinks/year but abstinent in previous year])

Table S4B：Multivariable logistic regression results between TyG and chronic pain after multiple imputation

| Variables | Model1 |  | Model2 | | Model3 | | Model4 |  |  |
| --- | --- | --- | --- | --- | --- | --- | --- | --- | --- |
|  | OR (95%CI) | p-value | OR (95%CI) | p-value | OR (95%CI) | p-value | OR (95%CI) | p-value |  |
| TyG index |  |  |  |  |  |  |  |  |  |
| Q1 | Ref |  | Ref |  | Ref |  | Ref |  |  |
| Q2 | 1.24(0.87,1.78) | 0.230 | 1.22(0.85,1.75) | 0.270 | 1.25(0.80,1.93) | 0.250 | 1.14(0.80,1.63) | 0.430 |  |
| Q3 | 1.32(0.93,1.87) | 0.110 | 1.28(0.92,1.78) | 0.140 | 1.23(0.81,1.88) | 0.260 | 1.14(0.81,1.59) | 0.430 |  |
| Q4 | 1.37(0.89,2.11) | 0.140 | 1.31(0.87,1.96) | 0.190 | 1.35(0.76,2.38) | 0.230 | 1.13(0.74,1.72) | 0.550 |  |
| Q5 | 1.97(1.34,2.91) | 0.001 | 1.91(1.34,2.74) | 0.001 | 1.86(1.07,3.22) | 0.030 | 1.58(1.09,2.31) | 0.020 |  |
| P for trend |  | 0.003 |  | 0.003 |  | 0.040 |  | 0.030 |  |

Note: TyG, triglyceride-glucose; OR, odds ratio; CI, confidence intervals.

Model1: Unadjusted

Model2: Adjusted for age, sex, education

Model3: Adjusted for age, education, sex, cardiovascular disease (CVD), Hyperlipidemia, albumin, neutrophil–lymphocyte ratio (NLR), serum iron, hemoglobin (HB), smoke status, alcohol status, cancer, anemia, chronic kidney disease (CKD), body mass index (BMI), diabetes mellitus (DM).

Model4: Adjusted for age, education, sex, neutrophil–lymphocyte ratio (NLR), smoke status, cancer, chronic kidney disease (CKD), body mass index (BMI), diabetes mellitus (DM).

TableS4C: Multivariable Cox regression results between TyG and all-cause mortality in participants with chronic pain after multiple imputation

| Variables | Model1 |  | Model2 | | Model3 | | Model4 |  |
| --- | --- | --- | --- | --- | --- | --- | --- | --- |
|  | HR (95%CI) | p-value | HR (95%CI) | p-value | HR (95%CI) | p-value | HR (95%CI) | p-value |
| TyG index |  |  |  |  |  |  |  |  |
| T1 | Ref |  | Ref |  | Ref |  | Ref |  |
| T2 | 2.01(1.39,2.90) | <0.001 | 1.23(0.87,1.75) | 0.243 | 1.32(0.91,1.93) | 0.144 | 1.30(0.89,1.91) | 0.177 |
| T3 | 2.68(1.86,3.87) | <0.001 | 1.65(1.15,2.38) | 0.007 | 2.10(1.39,3.16) | <0.001 | 1.95(1.41,2.69) | <0.001 |
| P for trend |  | <0.001 |  | 0.005 |  | <0.001 |  | <0.001 |

Note: TyG, triglyceride-glucose; HR, Hazard ratio; CI, confidence intervals.

Model1: Unadjusted

Model2: Adjusted for age, sex, education

Model3: Adjusted for age, education, sex, cardiovascular disease (CVD), Hyperlipidemia, albumin, neutrophil–lymphocyte ratio (NLR), serum iron, hemoglobin (HB), smoke status, alcohol status, cancer, chronic kidney disease (CKD), body mass index (BMI), diabetes mellitus (DM).

Model4: Adjusted for age, education, sex, cardiovascular disease (CVD), albumin, hemoglobin (HB), neutrophil–lymphocyte ratio (NLR), alcohol status, chronic kidney disease (CKD), body mass index (BMI), diabetes mellitus (DM).
