# Supplementary material for: The Mediating Role of WBC in the Relationship Between Triglyceride–Glucose Index and Chronic Pain: Evidence From NHANES 2001–2004 Data
Source: Pain Res Manag. 2026 Apr 21;2026:3793191. doi: 10.1155/prm/3793191 (PMC13096791; doi:10.1155/prm/3793191)
Supplement: Supplementary file 5 — Supporting Information 5 Figure S1: Mediation analyses: mediation effects of the neutrophil–lymphocyte ratio (NLR) on the TyG index–chronic pain (CP) relationship. [file PRM-2026-3793191-s005.docx]

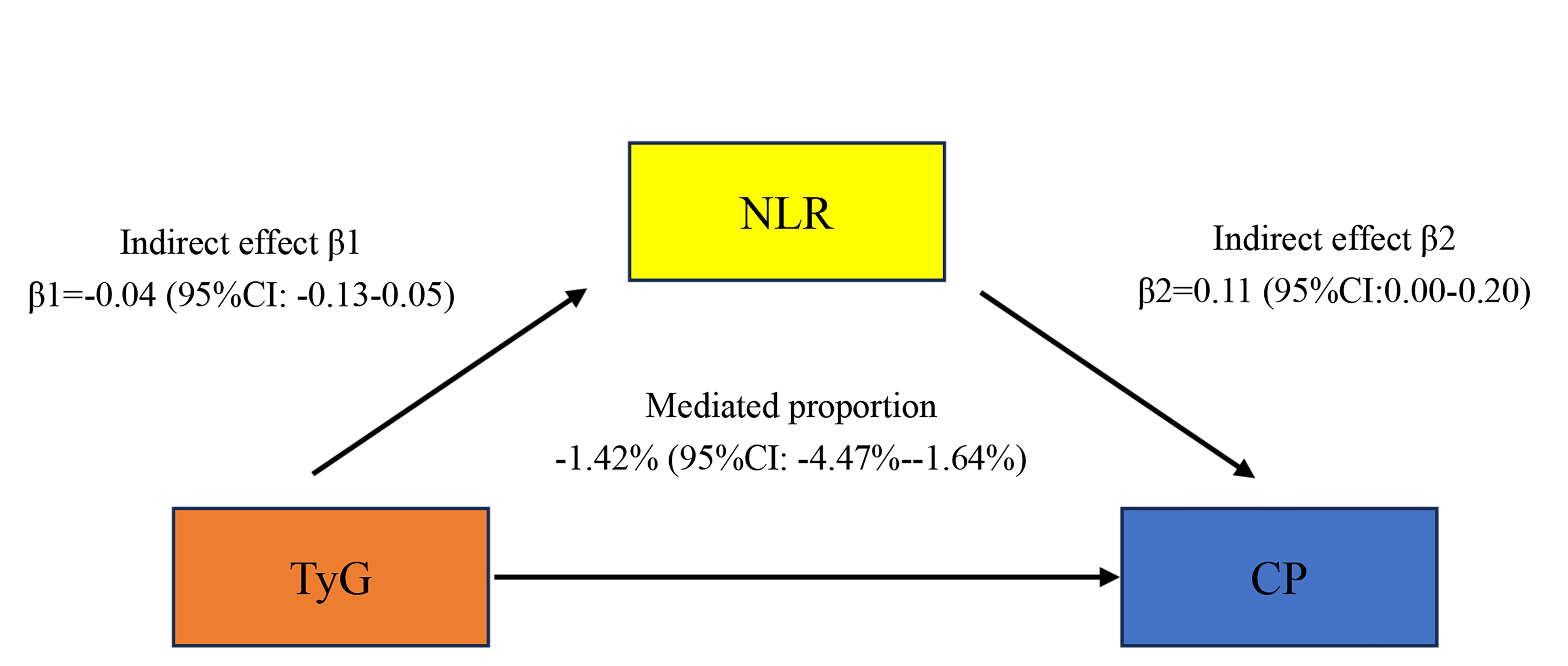


Figure S1 Mediation analyses: mediation effects of neutrophil–lymphocyte ratio (NLR) on the TyG index-chronic pain (CP) relationship
